# Examining Capacity and Functioning of Bicycle Coalitions: A Descriptive Study

**DOI:** 10.3389/fpubh.2017.00296

**Published:** 2017-11-08

**Authors:** Melissa Bopp, Dangaia Sims, Nicole Vairo, Emily Hentz-Leister

**Affiliations:** ^1^Department of Kinesiology, Pennsylvania State University, University Park, PA, United States

**Keywords:** bicycle, community, health promotion, advocacy, coalition

## Abstract

**Background:**

Bicycle coalitions represent a strong partner in creating bike-friendly communities through advocacy for physical infrastructure, encouragement for biking, or education about safety. Despite their versatility, little is known about their functioning. Therefore, the purpose of this study was to examine capacity, strengths, and weaknesses of these organizations.

**Methods:**

Bicycle coalitions/advocacy groups from English-speaking countries were recruited to take part in an online survey *via* email invitation. The survey addressed basic information about the coalition (community demographics, location), leadership, communication strategies, coalition priorities, barriers to programming/activities, and partners.

**Results:**

Coalitions (*n* = 56) from four countries completed the survey. Most coalitions operated as a non-profit (*n* = 44, 95.7%), 45% (*n* = 21) have paid staff as leaders, while 37% (*n* = 17) have volunteers as leaders. The following skills were represented in coalitions’ leadership: fundraising (*n* = 31, 53.4%), event planning (*n* = 31, 53.4%), urban planning (*n* = 26, 44%), and policy/legislation expertise (*n* = 26, 44.8%). Education (*n* = 26, 63.4%) and encouragement (*n* = 25, 61.6%) were viewed as top priorities and the safety of bicyclists (*n* = 21, 46.7%) and advocacy for infrastructure and policy (*n* = 22, 48.9%) is the focus of most activities. A lack of financial resources (*n* = 36, 81.8%) and capable personnel (*n* = 25, 56.8%) were significant barriers to offering programming in the community and that the availability of grants to address issues (*n* = 38, 86.4%) would be the top motivator for improvements.

**Conclusion:**

Bike coalitions represent a critical partner in creating activity-friendly environments and understanding their capacity allows for creating skill/capacity building intervention programs, development of effective toolkits and fostering strong collaborations to address physical inactivity.

## Introduction

Bicycling has the potential to improve rates of active travel and result in many positive health benefits. There is evidence showing that participation in regular biking for transportation is associated with decreased morbidity and mortality. A review by Hamer and Chida (1) indicated a decreased risk for cardiovascular diseases/events among active travelers and Saunders et al. (2) review found evidence suggesting that longer periods and distances of AT lead to reduced risk of diabetes. In addition to the notable health benefits, shifting trips from automobiles to bicycle or walking, could lead to a decrease in gasoline consumption, impacting fuel demand and prices. Rails to Trails Conservancy (3) estimates that a mode share shift to more active modes of travel could save 4–23 million tons of carbon a year for trips of less than 3 miles (4.83 km). Substantial economic benefits for communities are associated with greater biking, considering both direct costs (healthcare savings, time saved, recreational benefits) and indirect costs (real estate values, spending by bikers, fuel savings, jobs created, and return on infrastructure investment) (4, 5).

Bicycling is recognized as an important means for promoting public health, with participation varying significantly globally; rates in the United States are minimal, with less than 1% of trips taken *via* bicycle while in Western Europe rates of bicycling can exceed 30% of all trips (6–8). The US Department of Health and Human Services Healthy People 2020 initiative has set forth a goal to increase the proportion of trips made by biking among both adults and children, indicating that improvement of this behavior is an outcome of interest (9).

Given the extensive benefits, many communities may organize efforts to increase biking. Previous research has indicated that individuals are more likely to walk or bike if they identify their community as pedestrian or bicycle-friendly, perceive community level supports for biking, see others biking and perceive a “bike culture” (10–14). One community resource that can facilitate these is a bicycle coalition or advocacy group. The League of American Bicyclists (15) indicates that advocacy groups or coalitions help communities in several ways including: promoting awareness of issues related to biking, providing education for bicycle safety and skills, and creating accountability for the community in regards in cycling. This suggests that this community entity can have a widespread impact on bicycling in a region.

Despite their known utility, bicycle advocacy groups or coalitions have not been well studied. There is limited research addressing how these organizations function, their capacity or role within the larger community. Therein the purpose of this study was to examine an international sample of bicycle coalitions to document their programming, leadership structure and capacity and priorities. A stronger understanding of the inner working of bicycle coalitions to develop programs and training to improve their capacity and increase bicycling in communities.

## Materials and Methods

### Design

This was a cross-sectional, online survey (Qualtrics, Provo, UT, USA) conducted with a volunteer sample of representatives of bicycle advocacy groups and coalitions (herein referred to as coalitions). Data were collected July to September 2016. This study was approved by the Institutional Review Board at Pennsylvania State University.

### Participants and Recruitment

Lists of bicycle advocacy groups and coalitions in English-speaking countries were gathered from online sources (e.g., Alliance for Biking and Walking, League of American Bicyclists, etc.) and were used to locate the websites of bicycle coalitions. Where possible, leadership of the organization was identified and if an email address was available the individual was contacted *via* email with an invitation to participate in the survey, and reminder emails were sent 1 and 2 weeks after the initial email. Participants were provided a link to a website that would provide them with the complete survey prior to completing the survey to allow them an opportunity to see what kind of information was being sought and allow them to see input form others in their organization if necessary. Of all the possible coalitions, contact information for (*n* = 140) organizations were identified (*n* = 10), emails were returned as undeliverable for a final possible sample of (*n* = 130). Of those who started the survey (*n* = 61), 91.8% (*n* = 56) completed the survey for a final response rate of 43.1%.

### Measures

#### Description of the Community

Participants reported on the size of the community they served, if their community had a bike share, bike plan, or Complete Streets policy. Participants from the USA were asked whether their community was designated as a Bicycle Friendly Community by the League of American Bicyclists (15), how many League designated Bicycle Friendly Businesses and Universities were in the area they served. Using a 5-point Likert scale (1 = not at all supportive to 5 = very supportive), participants indicated the perceived level of political support for biking in their community.

#### Description of the Coalition and Partners

Representatives indicated how their organization functioned (as a community groups, a non-profit, or business), how their leadership was organized, the size of their leadership, number of paid staff members, primary source of funding, and membership in national biking advocacy groups. Coalitions were also provided with a list of possible community partners (e.g., parks and recreation department, schools) and were asked to indicate (yes/no) if they partner with or work with the entity to promote bicycling. Space was also provided for participants to list other organizations they worked with. Using a 5-point Likert scale (1 = never to 5 = always) coalitions indicated how frequently they worked with other biking coalitions, the purpose of the collaboration (yes/no; put on events, state or regional policy work, mentorship/guidance, other), if they would like to have facilitated connections with other coalitions (yes/no), and how the facilitated connection should happen (meetings, online community, webinars, other).

#### Coalition Priorities and Programming

Participants were asked to rank what their coalition spent the most time focusing on from: Engineering, Education, Encouragement, Enforcement and Evaluation. A list of important outcomes/benefits to the community was provided (sustainability issues, health outcomes, social outcomes, economic outcomes, biking for biking sake, awareness of local bike issues, decreased traffic, and congestion) and participants ranked their organizations top benefits/outcomes. A list of top priorities were provided for organizations to rank (safety/education, advocacy for environment and policy, social connections, serving as a voice for bikers in planning, addressing special populations). For all ranking questions, a ranking of one or two was reported on. A list of national programs was presented and participants could report (yes/no) whether their coalition participated in the program (Safe Routes to School, national Bike Challenge, Bike to work days/bike month, assistance to business for becoming bike friendly, Open Streets).

#### Perceived Barriers and Motivators for Programming

A list of possible barriers to programming was developed based on common challenges in volunteer/non-profit organizations (16). Participants ranked the barriers (lack of financial resources, lack of personnel/volunteers, lack of interest in the community, lack of physical infrastructure to support biking, unaware of appropriate strategies to use) and a ranking of one or two was reported on. Possible motivators to offering more programs were also listed (training for leadership/membership, incentives from outside organizations, partnerships with other organizations, availability of grant programs) and participants ranked the motivators.

#### Capacity, Consulting, and Grant-Writing

Participants were presented with a list of skills common in community/volunteer organizations and necessary for bicycle coalitions (16) and asked to report (yes/no) if their leadership had any background or expertise in: urban planning, traffic engineering, fundraising/development, event planning, legislation/policy development, public health, or research. Respondents indicated (yes/no) if their coalition had hired an outside consultant and if so, they were provided an open field to indicate what the consultants assisted with. On a 5-point Likert scale (1 = not at all capable to 5 = very capable) participants indicated their coalition’s capacity for grant writing and were given a slider scale to indicate how much grant funding the coalition had received in the past 5 years.

Communication strategies: respondents indicated (yes/no) what forms of common social media platforms (Facebook, Twitter, Instagram, and Snapchat) were used by the coalition. Participants indicated with a 5-point Likert scale (1 = not at all effective to 5 = very effective) how effective different forms of communication were for their organization (social media, emails/listserv, website, local news stories, and paid advertisements). An open field was provided to indicate the coalition’s greatest challenge with communication, responses were coded, and the frequency of responses was noted.

### Analysis

Basic descriptive statistics and frequencies described the sample. All analyses were conducted using SPSS 22.0 (IBM, Armonk, NY, USA).

## Results

A total of 56 coalitions completed the survey, with majority (*n* = 51, 91.1%) from the United States, with responses from Canada (*n* = 4, 7.1%) and New Zealand (*n* = 1, 1.8%). A description of the sample is found in Table 1. Coalitions predominately represented cities (*n* = 17, 30.4%) or regions (*n* = 17, 30.4%) in larger communities (greater than 1 million; *n* = 22, 39.3%). The majority represented a Bicycle Friendly Community and reported the presence of Bicycle Friendly Businesses and Universities. Most coalitions reported a number of supports for biking in their community, including bike shares, Complete Streets Policies, and bike plans.

**Table 1:** Description of the sample (*n* = 56).

	Mean	SD	*n*	%
**Community**
Size of community
<50,000 people			3	5.4
50,000–100,000 people			6	10.7
100,000–250,000 people			6	10.7
250,000–500,000 people			11	19.6
500,000–750,000 people			4	7.1
750,000–1,000,000 people			4	7.1
>1,000,000 people			22	39.3
Bike Friendly Community level
Bronze			17	37.8
Silver			8	17.8
Gold			5	11.1
Platinum			2	4.4
Number of Bike-friendly business	12.95	16.38		
Number of Bike-friendly universities	2.54	0.69		
Community has a bike share			27	51.9
Complete Streets Policy for the community
None			15	29.4
Ordinance			9	17.6
Resolution			8	15.7
Master plan			15	29.4
Has, unsure of type			4	7.8
Community has a bike plan			43	87.8
**Coalition**				
Area representing				
City/town			17	30.4
County			9	16.1
Region			17	30.4
State/province			13	23.2
Operation				
As a community group			1	2.2
As a non-profit organization			54	95.7
As a for-profit business			1	2.2
Leadership organization				
Paid staff			21	45.7
Community volunteers			17	37
Elected by the coalition			6	13
Combination of paid and volunteer			2	4.3
Size of coalition leadership	10.22	4.82		
Number of paid staff members	5.86	5.61		
Primary source of funding				
Dues			10	21.7
Grants/contracts			19	41.3
Gifts/donations			8	17.4
Sponsorship			1	2.2
Government			2	4.3
Events			2	4.3
Sales			4	8.7
Membership in national biking advocacy groups				
Rails to Trails Conservancy			9	15.5
The League of American Bicyclists			38	65.5
Alliance for Biking and Walking			32	55.2
Association of Pedestrian and Biking Professionals			10	17.2

Most of the coalitions reported operating as a non-profit (*n* = 54, 95.7%), with the number of paid staff ranging from 0 to 20 (mean = 5.86 ± 5.61). Coalitions were led predominately by paid staff (*n* = 21, 45.7%) and community volunteers (*n* = 17, 37%), with an average leadership of 10.22 ± 4.82 individuals. Grants/contracts (*n* = 19, 41.3%) and dues from members (*n* = 10, 21.7%) were the most commonly reported primary source of funding. Most coalitions reported membership in national bicycle advocacy-related organizations. Only *n* = 18 (40%) perceived a somewhat or very favorable political support for biking in their community.

### Coalition Programs, Priorities, and Partners

The coalitions’ programs and priorities are reported in Table 2. Education (*n* = 26, 63.4%) and encouragement (*n* = 25, 61%) were the areas where coalitions reported focusing their efforts on, while coalitions reported social outcomes (*n* = 22, 48.9%) were the most important outcome or benefit for the community. Respondents identified “serving as a voice for bikers in urban planning” (*n* = 23, 51.1%) advocating for supportive environment and policy (*n* = 22, 48.9%) and safety/education (*n* = 21, 46.7%) as their main priorities. Most coalitions reported engaging in some national/international programs, with Bike to work days/month (*n* = 42, 72.4%) and Safe Routes to School (*n* = 31, 53.4%) as the most common. Most (*n* = 31, 70.5%) reported offering assistance to local businesses looking to become bicycle friendly. Coalitions noted a number of barriers to programming (Table 3), including a lack of financial resources (*n* = 36, 81.8%) and a lack of personnel/volunteers (*n* = 25, 56.8%). Most indicated that the availability of grant programs (*n* = 38, 86.4%) and incentives from outside organizations (*n* = 19, 43.2%) would be motivating to offer more programming. A wide range of community partners were noted for coalitions, including parks and recreation departments (*n* = 37, 66.1%), schools (*n* = 35, 62.5%), community biking groups (*n* = 34, 60.7%), local media (*n* = 34, 60.7%), transit organizations (*n* = 28, 50%), public health departments (*n* = 26, 46.4%), health-related community groups (*n* = 24, 42.9%), pedestrian groups (*n* = 24, 42.9%), bicycle friendly businesses (*n* = 23, 41.1%), universities (*n* = 19, 33.9%), hospitals (*n* = 21, 37.5%), and YMCAs (*n* = 14, 25%). Collaborations with other bicycle coalitions was moderate; 18 (40%) reported collaboration at least some of the time, with regional work (*n* = 32, 57.1%), collaboration for events (*n* = 22, 39.3%), or mentorship (*n* = 15, 26.8%) as the most common reasons for collaboration. Half (*n* = 28) indicated a desire for some form of facilitation for connections with other coalitions, *via* meetings (*n* = 10, 38.5%), an online community (*n* = 10, 38.5%), or webinars (*n* = 5, 19.2%).

**Table 2:** Coalition programs and priorities (*n* = 56).

	*n*	%
What the coalition does the most work for		
Engineering	23	56.1
Education	26	63.4
Encouragement	25	61
Enforcement	7	17.1
Evaluation	7	17.1
Most important outcomes/benefits for the community		
Sustainability issues	9	20
Health outcomes	17	42.2
Social outcomes	22	48.9
Economic outcomes	18	40
Biking for biking’s sake	7	15.6
Awareness of local bike issues	8	17.8
Decreased traffic and congestion	7	15.6
Top priorities		
Safety/education	21	46.7
Advocacy for environment and policy	22	48.9
Encouragement for biking in your community	17	37.8
Socially connecting bikers	2	4.4
Serving as a voice for bikers in urban planning	23	51.1
Addressing concerns for special populations (e.g., children, older adults)	5	11.1
Program participation		
Safe Routes to School	31	53.4
Bike challenge	29	50
Bike to work days/week	42	72.4
National bike month	33	56.9
Assistance for businesses to become bicycle friendly	31	70.5
Open Streets	21	36.2

**Table 3:** Coalition perceived barriers and motivators for programming (*n* = 56).

	*n*	%
Barriers		
Lack of financial resources	36	81.8
Lack of personnel/volunteers	25	56.8
Lack of community interest	12	27.3
Lack of physical infrastructure to support biking	16	36.4
Unaware of appropriate strategies to use	6	13.6
Motivators		
Training for leadership/members	13	29.5
Incentives from outside organizations	19	43.2
Partnerships with other organizations	18	40.9
Availability of grant programs	38	86.4

### Capacity, Consulting, and Grant-Writing

Within the coalition’s leadership, the reported skillset included: fundraising (*n* = 31, 55.4%), event planning (*n* = 31, 55.4%), urban planning (*n* = 26, 46.4%), legislation/policy development (*n* = 26, 46.4%), public health (*n* = 22, 39.3%), research/evaluation (*n* = 18, 32.1%), and transportation engineering (*n* = 9, 16.1%). Coalitions reported that they were somewhat capable with grant writing; 22 (49.5%) reported they were somewhat or very capable, while 12 (27.3%) indicated they were not capable at all. Most reported receiving some funding in the past 5 years for funding (range: $0–250,000), with a mean of $146,730 ± 105.69. The majority (*n* = 29, 64.4%) reported hiring an outside consultant with strategic planning (*n* = 16, 55.1%) as the main cause of seeking assistance.

### Communication and Outreach

Coalitions’ communication strategies are outlined in Table 4. All respondents reported using social media as a form of communication, with Facebook (*n* = 45, 77.6%) and Twitter (*n* = 38, 65.5%) as the most common strategies. Overall, coalitions didn’t perceive their communication methods were that effective; emails/listserv and social media had higher perceived effectiveness compared with local news sources, their website, and paid advertisements. The largest reported challenge (*n* = 26, 44.8%) associated with communication was related to time and effort directed toward this form of outreach.

**Table 4:** Coalition communication strategies (*n* = 56).

	Mean	SD	*n*	%
Social media use				
Facebook			45	77.6
Twitter			38	65.5
Instagram			26	44.8
Snapchat			2	3.4
Perceived effectiveness for communication methods				
Social media	3.48	0.89		
Emails/listserv	3.52	0.92		
Website	3.05	0.87		
Local news source	3.4	0.9		
Paid advertisements	2.38	0.87		

## Discussion

Bicycle coalitions and advocacy groups serve as an important partner on the frontline of efforts to promote bike-friendly communities. This study is among the first to provide insight on the capacity, priorities and perceptions of a broad sample of organizations. Findings suggest that organizations have moderate success but may lack capacity for more extensive programming with even greater reach. The information garnered here can serve as a foundation for further research on these types of organizations as well as effective programming to help them advance the mission of safer bicycling for all.

Previous research has indicated that the role of citizen-driven community health organizations is essential for impacting behavior and associated health outcomes (17, 18). The capacity of these organizations can often drive the success or failure of efforts, for example in the Bootheel Heart Health project which used a community-based coalition to drive programming and activities to prevent cardiovascular disease, communities with a stronger coalition had more success in changing disease risk factors (physical inactivity, smoking, cholesterol screening) (18). This underscores the importance of understanding how coalitions function in order to maximize their impact. In the present study, many coalitions indicated they had strengths among their members for fundraising and event planning but lacked capacity in grant writing and research/evaluation. This presents an opportunity for training; programs could be built to deliver biking coalition-specific content and assist with building skills in these areas to members of these organizations. Webinars or webcasts would provide an opportunity for representatives from organizations across a broad geographic area to participate and minimize operational costs.

Like many community organizations, these coalitions indicated that they struggled to provide programming as a result of lack of volunteers and lack of funding. The Centers for Disease Control and Prevention’s guide for community coalitions (19) recommends recruiting volunteers and members to work on specific issues, programs or policies. For bicycle coalitions, this may be an effective strategy; gathering interested parties for specific activities to increase participation from the community rather than drawing from a small pool of dedicated volunteers or members who may be overburdened. Additionally, the CDC recommends partnering with organizations that have similar or parallel missions in order to be more effective. Although the organizations in the current study reported having a wide range of community partner organizations, it may be worthwhile to consider non-traditional partners who may see additional value in bike-friendly communities. For example, sustainability-focused groups are increasingly focused on walking and biking as environmentally friendly modes of travel and may serve as a strong advocate to help with decreasing pollution. Groups focused on air quality or respiratory disease prevention may also serve as a partner in line with this mission.

The main priorities noted by the majority of organizations were related to engineering and education. Significant research has indicated the importance of supportive physical infrastructure to support biking, especially among youth, women and older adults (12, 14, 20–22). This highlights the importance of advocacy groups to help in creating and maintaining safe spaces for people to bike. Supportive policies, such as Complete Streets also contribute to the development and maintenance of supportive infrastructure. As of 2016 in the United States, 1,232 Complete Streets policies have been adopted, with a sharp increase since 2011. In the current study, approximately 70% of organizations reported their area has a Complete Streets policy, with part of a master plan as the most common response, which indicates a greater commitment than national trends, where a resolution is the most common policy (23). Bicycle coalitions often play a role in bicycle planning with local governments, providing insight and experience from the streets. The Alliance for Walking and Biking (24) has indicated that those communities with more active advocacy groups have lower rates of bicycle injury and fatalities, showing the preventive role that advocacy groups can have. For education, the bulk of the literature indicates that bicycle safety educational campaigns have been primarily aimed at youth (25), building road safety skills and encouraging helmet use. Some campaigns, based in worksites have focused on building road riding skills for adults and have seen moderate success (26). These two main priorities have significant implications for bicycling in communities and rightfully warrant the bulk of attention of advocacy groups.

One of the other significant challenges these organizations indicated were those associated with effective communication. The World Health Organization (WHO) notes the importance of communication in health promotion efforts, noting its role in effective interventions and efforts to target behavior (27). WHO recognizes some of the challenges associated with communication, especially for reach with a message for health promotion, noting the importance of effective message framing and using different channels. Many of the organizations indicated that they used social media and this was an effective form of communication for them. Recent research has outlined how social media can be used in health promotion efforts and communication for health-related programming, though reach and outcomes are still unclear (28, 29). A recent campaign to promote active travel at a large university used social media extensively and saw widespread reach and an increased likelihood of active travel among those who had been exposed to the social media efforts (30, 31). This indicates that social media may be evolving to become among the more far-reaching forms of communication in biking-related initiatives. Further training on social media use and communication strategies could be developed and offered for bicycle coalitions.

Despite the insightful findings in the current study there were a number of limitations. The method of recruitment (online, *via* websites) could have resulted in a biased, volunteer sample, with access limited to only those organizations with available contact information. Additionally, we relied on self-report measures for our main outcomes, which could be subject to recall bias or social desirability. Lastly, although our response rate was adequate for online surveys, we were unable to determine any characteristics of non-responders and therein if our participants were representative of our larger target population. We were able to determine that response rates were slightly better among USA participants and if we had an email address of an actual contact within the organization rather than those who had a generic “information” email address available. This gives insight for further studies looking to address the work of bicycle coalitions.

These limitations notwithstanding, this study provides important insight into how bicycle coalitions function, their capacity and their priorities. These organizations remain one of the most important resources in the quest to make our communities more bicycle-friendly. Public health practitioners looking to partner with bicycle coalitions can gain insight from this study, notably building programming around their strengths in encouraging cycling and advocacy and outreach. Other strategies for partnership could include building community wide initiatives with other organizations that help to bridge gaps in capacity as noted in this study (e.g., grant writing). These findings provide ample avenues for further investigation as well as a direction for initiatives aimed at coalitions. Enhancing their function can only result in greater participation in biking in communities, with the long-term outcomes of improved population level health as well as many notable environmental and economic benefits.

## Ethics Statement

This study was carried out in accordance with the recommendations of the Institutional Review Board at Pennsylvania State University with written informed consent from all subjects. All subjects gave written informed consent in accordance with the Declaration of Helsinki. The protocol was approved by the the Institutional Review Board at Pennsylvania State University.

## Author Contributions

MB designed the study, oversaw data collection, completed analyses and drafted the manuscript. DS assisted in study design and data collection and provided feedback on the manuscript. NV and EH assisted in data collection and provided feedback on the manuscript.

## Conflict of Interest Statement

The authors declare that the research was conducted in the absence of any commercial or financial relationships that could be construed as a potential conflict of interest.
